# An Updated Review on Imaging and Staging of Anal Cancer—Not Just Rectal Cancer

**DOI:** 10.3390/tomography9050135

**Published:** 2023-09-04

**Authors:** Alessio Congedo, Davide Mallardi, Ginevra Danti, Federica De Muzio, Vincenza Granata, Vittorio Miele

**Affiliations:** 1 Department of Radiology, Careggi University Hospital, Largo Brambilla 3, 50134 Florence, Italy; alessio.congedo@gmail.com (A.C.); mallardidav@gmail.com (D.M.); vmiele@sirm.org (V.M.); 2 Department of Medicine and Health Sciences V. Tiberio, University of Molise, 86100 Campobasso, Italy; demuziofederica@gmail.com; 3 Division of Radiology, Istituto Nazionale Tumori IRCCS Fondazione Pascale—IRCCS di Napoli, 80131 Naples, Italy; v.granata@istitutotumori.na.it

**Keywords:** anal canal, anal cancer, rectal cancer, staging, TNM

## Abstract

Anal cancer is a rare disease, but its incidence has been increasing steadily. Primary staging and assessment after chemoradiation therapy are commonly performed using MRI, which is considered to be the preferred imaging modality. CT and PET/CT are useful in evaluating lymph node metastases and distant metastatic disease. Anal squamous-cell carcinoma (ASCC) and rectal adenocarcinoma are typically indistinguishable on MRI, and a biopsy prior to imaging is necessary to accurately stage the tumor and determine the treatment approach. This review discusses the histology, MR technique, diagnosis, staging, and treatment of anal cancer, with a particular focus on the differences in TNM staging between anal and rectal carcinomas. Purpose: This review discusses the histology, MR technique, diagnosis, staging, and treatment of anal cancer, with a particular focus on the differences in TNM staging between anal squamous-cell carcinoma (ASCC) and rectal adenocarcinoma. Methods and materials: To conduct this updated review, a comprehensive literature search was performed using prominent medical databases, including PubMed and Embase. The search was limited to articles published within the last 10 years (2013–2023) to ensure their relevance to the current state of knowledge. Inclusion criteria: (1) articles that provided substantial information on the diagnostic techniques used for ASCC, mainly focusing on imaging, were included; (2) studies reporting on emerging technologies; (3) English-language articles. Exclusion criteria: articles that did not meet the inclusion criteria, case reports, or articles with insufficient data. The primary outcome of this review is to assess the accuracy and efficacy of different diagnostic modalities, including CT, MRI, and PET, in diagnosing ASCC. The secondary outcomes are as follows: (1) to identify any advancements or innovations in diagnostic techniques for ASCC over the past decade; (2) to highlight the challenges and limitations of the diagnostic process. Results: ASCC is a rare disease; however, its incidence has been steadily increasing. Primary staging and assessment after chemoradiation therapy are commonly performed using MRI, which is considered to be the preferred imaging modality. CT and PET/CT are useful in evaluating lymph node metastases and distant metastatic disease. Conclusion: ASCC and rectal adenocarcinoma are the most common histological subtypes and are typically indistinguishable on MRI; therefore, a biopsy prior to imaging is necessary to stage the tumor accurately and determine the treatment approach.

## 1. Introduction

Anal cancer is relatively rare, comprising only approximately 2.5% of gastrointestinal tumors; however, the incidence of anal cancer has been steadily increasing in recent years, with a notable rise observed over the past three to four decades.

The rectum and the anal canal are structurally connected. The rectum begins at the point where the taenia converge and extends to the upper border of the puborectalis muscle; the anal canal starts at the upper border of the puborectalis muscle and extends to the anal verge. Rectal and anal cancers are different types of tumors. Adenocarcinomas are the most common form of rectal cancer, whereas anal cancers are generally squamous-cell carcinomas (SCCs) that arise from the squamous mucosa of the anal canal [1,2,3]. The TNM staging also varies between the two types of cancer. The T1–T3 stages for anal cancer are based on the size, while for rectal cancer they are based on the depth of invasion. The N stage is determined by the location of positive lymph nodes in anal cancer, and by the number of positive lymph nodes in rectal cancer. In terms of treatment, most patients with rectal cancer undergo surgery. On the other hand, almost all patients with anal cancer receive chemotherapy and radiation therapy, with surgery reserved for the early stage (T1N0) [2,4,5]. 

## 2. Clinical Features and Epidemiology

Anal cancer is more prevalent among women than men and is most frequently diagnosed between the ages of 45 and 75 years [4]. SCCs are the most common type of anal cancer; other rare types include adenocarcinomas and neuroendocrine tumors [5].

Risk factors for developing anal cancer include persistent human papillomavirus (HPV) infection (particularly serotypes 16 and 18), immunosuppression caused by HIV infection or occurring after organ transplantation, receptive anal intercourse, a history of cervical or vulvar cancer, and cigarette smoking [6].

There has been a noticeable increase in ASCC cases in several populations, particularly in the Americas, Northern and Western Europe, and Australia. Among the various histological subtypes, ASCC is more prevalent in these regions and largely contributes to the overall increase in the number of ASCC cases. However, Asian and Central/Eastern European populations have reported lower rates of ASCC and have seen limited changes over time. In contrast, the incidence of anal adenocarcinoma (AAC) has generally remained stable or has decreased in most populations. The exact reasons for the increasing ASCC incidence rates in diverse populations are not entirely clear, but they likely result from changes in the prevalence of environmental risk factors [7].

One well-established risk factor for ASCC is human papillomavirus (HPV) infection, particularly HPV16. Persistent anal HPV infection plays a significant role in the potential development of ASCC. Factors contributing to this persistence include concurrent cervical HPV infection, receptive anal intercourse, younger age at first intercourse, and coexisting human immunodeficiency virus (HIV) infection. The increase in anal cancer rates in women in certain countries can likely be attributed to the rising prevalence of cervical HPV infection driven by significant changes in sexual behavior in recent decades. Interestingly, minimal changes have been observed in the incidence of females with ASCC in Asian countries, where the number of women’s sexual partners and the prevalence of cervical HPV infection have historically been lower than those in Europe or the Americas [6,7].

In both males and females, those with HIV infection experienced a higher occurrence of anal HPV infection and ASCC. As the duration of HIV infection extends, the risk of ASCC also escalates. Existing evidence indicates that the implementation of effective therapies to control HIV infection significantly increases the lifespan of HIV-positive individuals. Surprisingly, this increase in survival is associated with a rise in the incidence of ASCC. Several studies have also established a correlation between tobacco smoking and ASCC [7].

Most patients present with a range of symptoms, including bleeding, anorectal pain, and sensation of mass; other clinical manifestations include perianal pruritus, incontinence, and changes in bowel movements. It is not uncommon for patients to initially seek medical attention for symptoms resembling those of benign conditions, such as hemorrhoids, leading to a delay in diagnosis. In some instances, patients may not exhibit any symptoms at all [5,8].

## 3. Histology

**Figure 1:**
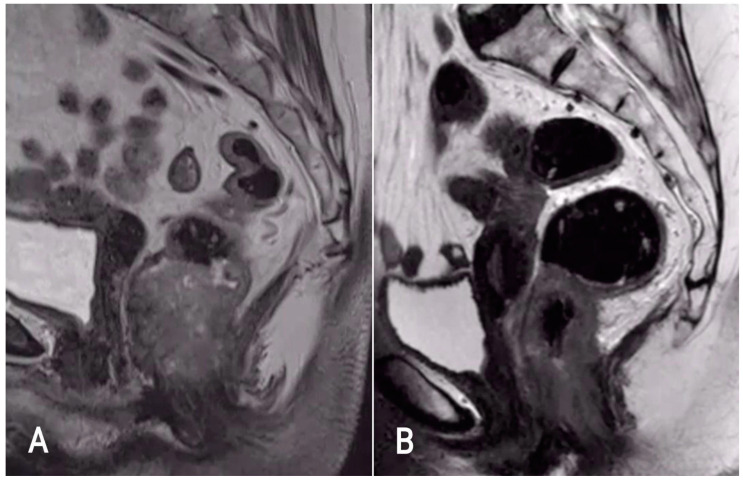
Sagittal T2-weighted MR images of rectal adenocarcinoma (**A**) and anal squamous-cell carcinoma (**B**). Both typically display intermediate signal intensity on T2-weighted imaging.

Anal and rectal cancers are often indistinguishable on magnetic resonance imaging (MRI). Both ASCC and rectal adenocarcinoma typically display intermediate signal intensity on T2-weighted imaging and show signs of diffusion restriction on diffusion-weighted imaging (DWI) (Figure 1). This can make it challenging to differentiate between the two types of cancer on routine MRI. Rectal adenocarcinomas may extend into or originate primarily from the anal canal, and ASCCs may extend into the rectum or present with skip lesions higher in the rectum [2,4]. Therefore, the location of the tumor does not provide information regarding its histological type. It is necessary to perform a biopsy prior to MRI to determine the appropriate staging and treatment approach.

## 4. Anatomy of the Anal Canal Location

The anal canal is approximately 3.5 to 4 cm in length and can be divided into anatomic and surgical canals. The anatomic canal extends from the dentate or pectineal line to the anal verge. The surgical canal is considered to be slightly longer and extends from the anorectal junction to the anal verge [8]. Despite not being visible on MRI, the location of the dentate line can be approximated by dividing the anal canal into thirds. It is situated at the junction of the upper one-third and lower two-thirds of the canal. The upper portion is covered with a transitional zone or rectal glandular mucosa, whereas the lower part is covered with non-keratinizing squamous epithelium [9,10].

The anal sphincter complex consists of the internal anal sphincter (IAS) and the external anal sphincter (EAS). The IAS is an extension of the inner circular muscle layer of the rectal muscularis propria; the EAS is made up of closely related skeletal muscles, including the inferior portion of the levator ani, the puborectalis, and the external sphincter muscles. The IAS and EAS are separated by the intersphincteric plane, which consists of a fibro-fatty muscular layer [11,12]. 

**Figure 2:**
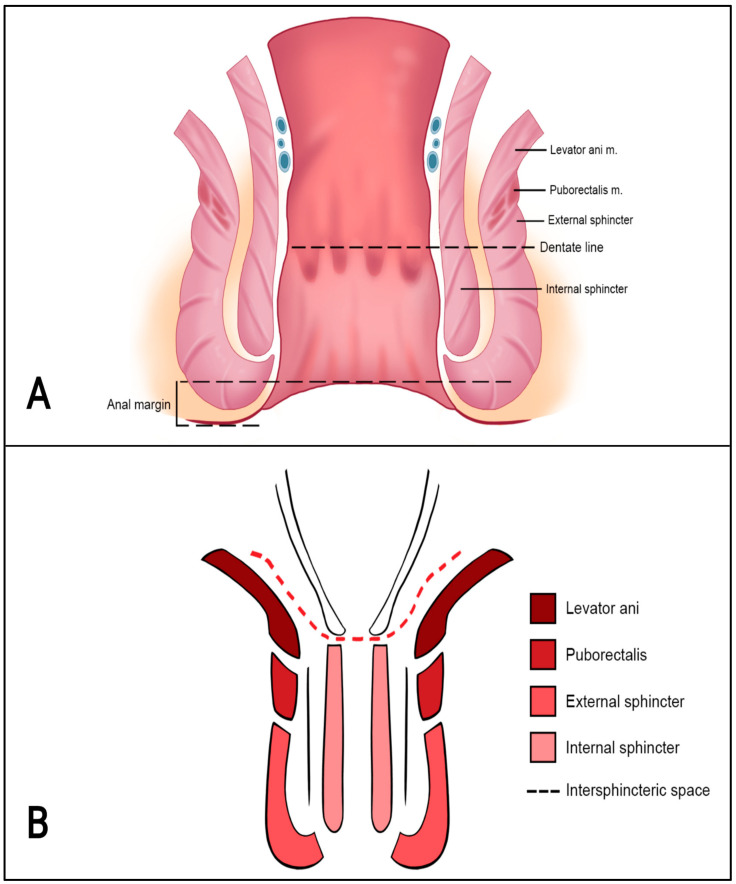
(**A**,**B**) Anatomy of the anal canal.

**Figure 3:**
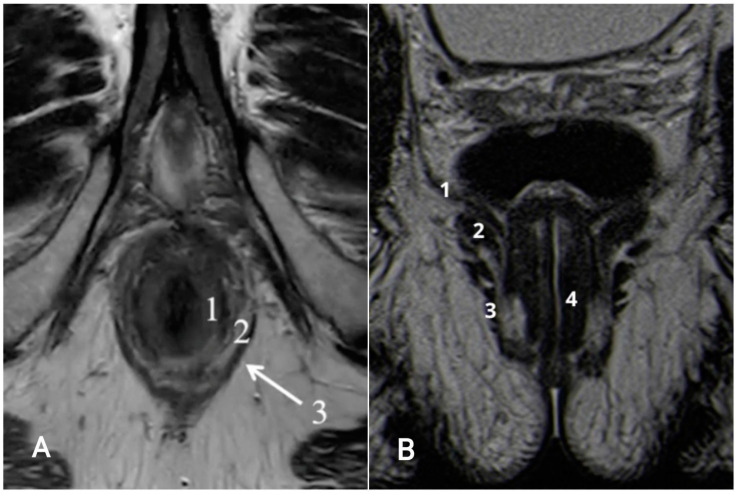
(**A**) Anatomy of the anal canal. T2-weighted axial MR image: 1 = internal sphincter (low–intermediate signal), 2 = fatty intersphincteric space (high signal), 3 = external sphincter (low signal, white arrow); (**B**) T2-weighted coronal MR image: 1 = levator ani muscle, 2 = puborectalis muscle, 3 = external sphincter, 4 = internal sphincter.

On MRI, the IAS is homogeneous and shows a low–intermediate T2 signal and intense enhancement post-contrast; the EAS appears striated and displays a lower T2 signal and post-contrast hypoenhancement compared to the IAS; the intersphincteric space appears hyperintense and does not show enhancement post-contrast [4] (Figure 2 and Figure 3).

## 5. MR Technique

High-resolution anal imaging is necessary to precisely evaluate the dimensions and location of anal cancer, as well as to determine the involvement of the sphincter complex and the relationship between the tumor and adjacent pelvic organs. For optimal results, a magnetic field intensity of no less than 1.5 T is recommended. Surface-based array coils are typically employed for this purpose. An endorectal coil cannot be employed because of the potential discomfort that it may induce. No specific patient preparation is typically required; however, anal canal distension with jelly may be helpful for visualizing small lesions [5].

To ensure a comprehensive evaluation, it is advisable to obtain ample coverage of both the anal margin and inguinal regions, encompassing the sacral promontory and the area immediately beneath the aortic bifurcation, to facilitate a more precise assessment of superior nodal stations [10].

**Figure 4:**
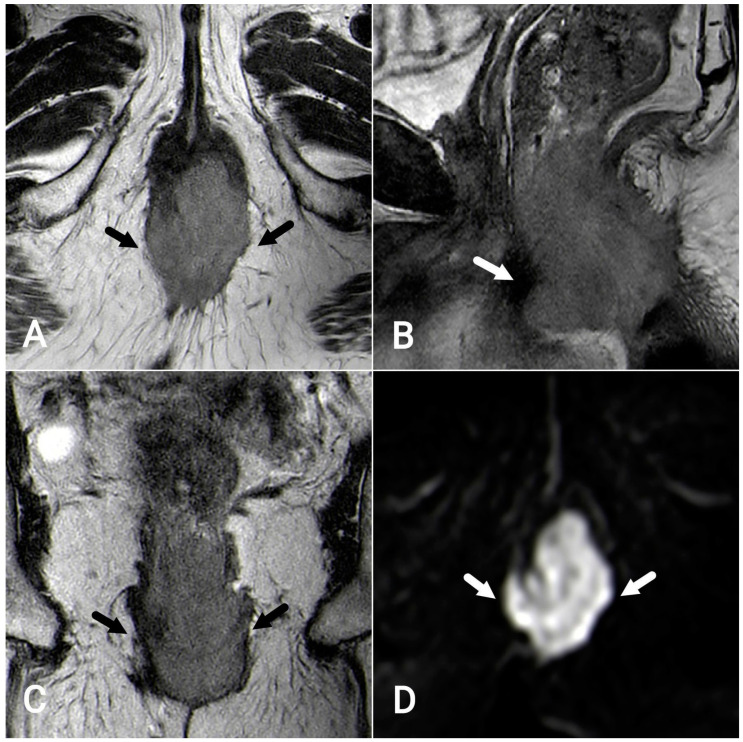
Squamous-cell carcinoma of the anal canal. Axial (**A**), sagittal (**B**), and coronal (**C**) T2-weighted MR images: heterogeneous lesion measuring 6.5 × 5.5 × 4 cm invading the left ischioanal fossa, internal and external sphincter muscles, and puborectalis muscle bilaterally. The tumor is characterized by restricted diffusion on diffusion-weighted imaging (**D**, white arrows).

High-resolution T2-weighted images in the axial, sagittal, and coronal planes are of utmost importance. The coronal images are aligned parallel to the long axis of the anal canal, whereas the axial images are oriented perpendicular to it. Usually, a slice thickness of less than 4 mm and a narrow field of view are employed to achieve optimal results [10,13]. Contrast-enhanced gradient T1-weighted imaging may improve the visualization of the tumor and its relationship with the sphincters, although this is optional. In most patients, high-resolution T2W images are sufficient to provide relevant information [4,14,15]. Diffusion-weighted imaging aids in the localization and characterization of tumors and lymph nodes [16,17] (Figure 4A–D).

Anal cancers show high signal intensity compared to skeletal muscles on T2-weighted images and low-to-intermediate signal intensity relative to ischioanal fat on T1-weighted images [10].

## 6. Diagnosis

The initial diagnostic evaluation of anal cancer includes digital rectal examination (DRE) and proctoscopy with biopsy [4,18].

According to joint guidelines from the European Society for Medical Oncology, the European Society of Surgical Oncology, and the European Society of Radiotherapy and Oncology (ESMO-ESSO-ESTRO), as well as guidelines from the American Society of Colon and Rectal Surgeons, MRI of the pelvis and endoanal ultrasound (EAUS) should be used for the standard management of anal cancer [19,20]. If MRI is not available, EAUS is recommended as an alternative. However, EAUS should be reserved for small T1 lesions because of its limited field of view, which may restrict the identification of regional lymph nodes [21,22,23].

CT of the chest, abdomen, and pelvis with intravenous contrast is commonly used to evaluate distant metastatic disease and lymphadenopathy. CT offers several advantages in the assessment of ASCC. First, its widespread availability in medical facilities makes it a practical and cost-effective option for initial tumor evaluation and staging. CT’s high spatial resolution enables detailed imaging of the pelvic region, facilitating visualization of the primary tumor and nearby lymph nodes, thus enabling anatomical delineation. Moreover, CT plays a crucial role in identifying bone metastases, which are relatively common in the advanced stages of ASCC. Additionally, CT scans are efficient and quick to perform, reduce examination time, and enhance patient comfort.

Despite its numerous benefits, clinicians should consider the limitations associated with CT. Soft-tissue contrast may pose challenges in accurately distinguishing tumor tissue from adjacent organs or structures, potentially leading to inaccuracies in tumor delineation. Furthermore, the utilization of ionizing radiation in CT raises concerns, especially in young people and patients undergoing repeated imaging or with a history of radiation exposure.

The 18F-FDG PET/CT technique exhibits sensitivity in detecting the primary tumor; however, its ability to fully characterize the tumor is limited.

The 18F-FDG PET/CT technique is currently recommended for radiation treatment planning because of its ability to detect nodal disease [4,24,25]. PET/CT is more sensitive than CT alone in identifying nodes; however, it has modest specificity, leading to false-positive findings in cases of inflammatory conditions. Moreover, PET/CT’s sensitivity might be lower than that of MRI in detecting perirectal nodes. However, this limitation does not affect management, because perirectal nodes are routinely included in the irradiated volume [9].

Additionally, PET has inherent limitations owing to the use of FDG, which is not a cancer-specific agent. This can lead to potential false-positive findings in cases involving infections, inflammatory conditions, postoperative scenarios, tumors with low glycolytic activity (such as small tumors), or diseases located near physiological uptake sites such as the heart, bladder, kidneys, or liver [9].

In recent years, the use of artificial intelligence (AI) and radiomics in healthcare has gained popularity for supporting oncological imaging; these technologies have potential applications in the detection and characterization of colorectal cancer, and in monitoring tumor response to treatment [26,27,28,29]. 

Radiomics is a computational technique that involves the extraction and analysis of vast amounts of quantitative data from medical images. By applying machine learning and other data-mining methods, radiomics can reveal previously unseen patterns and relationships within medical images, which can be used to predict patient outcomes, personalize treatment plans, and improve overall patient care [30,31,32,33,34,35,36,37,38].

## 7. Staging

TNM staging of anal cancer is based on the assessment of tumor size and local organ involvement (T), regional lymph node metastasis (N), and distant metastatic disease (M) [4,39].

### 7.1. T-Staging

**Figure 5:**
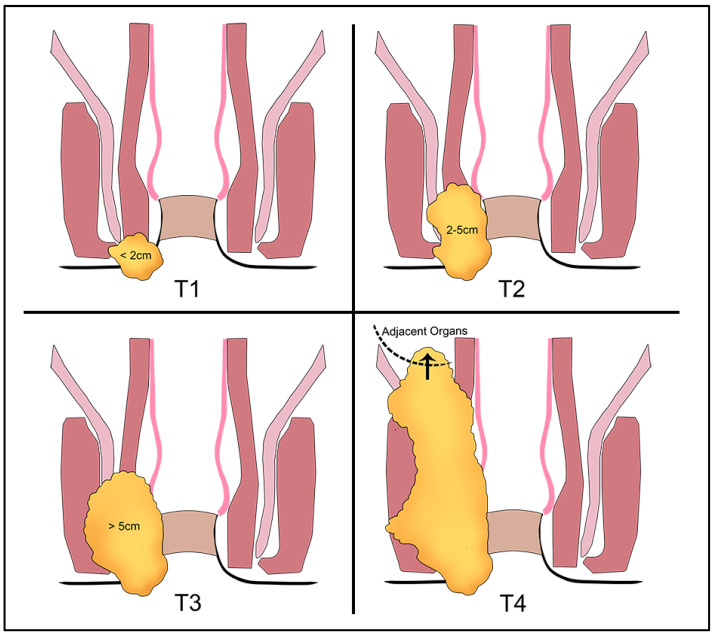
T-staging of anal cancer [41].

The T-stage classification for anal cancer is established through the assessment of the maximum diameter of the tumor, which distinguishes it from the T-stage classification for rectal cancer, which is based on the depth of invasion. In anal cancer, T1 tumors are less than 2 cm in diameter, T2 tumors are between 2 and 5 cm, T3 tumors are greater than 5 cm, and T4 tumors invade other organs or structures such as the prostate and seminal vesicles, penis, vagina, cervix/uterus, ovaries, ureters and urethra, bone, nerves and vessels, and striated muscles (e.g., pelvic sidewall) [40]. The European Society for Medical Oncology (ESMO) states that the T4 stage classification for anal canal carcinoma is not impacted by the invasion of the external and/or internal anal sphincter, puborectalis muscle, levator ani muscle, rectal wall, or perianal skin [10] (Figure 5).

It is important to provide information on both the axial and circumferential planes, as well as the craniocaudal plane. It is helpful to specify which layer of the anal canal is involved in the axial plane. This can help radiation oncologists to delineate the gross tumor volume (GTV) for radiotherapy planning [42].

### 7.2. N-Staging

Whole-body imaging is recommended to identify nodal or distant metastases. In particular, locoregional nodes are studied on MRI. The prognosis is significantly impacted by the presence of nodal invasion. For anal carcinomas, the 5-year survival rate is 76% for N0 disease and 53.5% for N1 nodal involvement [43]. The additional role of PET/CT and targeted ultrasound-guided fine-needle aspiration is also important for the confirmation of lymph node disease localization [44]. A meta-analysis conducted by Jones et al. showed that the use of 18F-FDG-PET resulted in upstaging in 15% of cases and downstaging in 15% of cases [45,46]. The diagnostic performance of 18F-FDG-PET may be improved in the future with the use of hybrid PET-MRI scanners, but additional research is needed before this can be considered as a standard of care [47].

Staging lymph nodes using MRI and CT scans can be difficult. The differences between malignant and benign lymph nodes for anal cancer have not been described in the literature, whereas they are for rectal cancer. Similar to rectal cancer, the common criterion used to identify malignant lymph nodes is a threshold of >9 mm in the short-axis diameter. However, assessing nodal involvement based only on size could lead to false-positive and false-negative results due to reactive lymph nodes and microscopic nodal involvement, respectively [4,48]. Because of the limitations of the dimensional criteria, it is essential to evaluate the MR nodal morphological characteristics associated with metastatic infiltration. The presence of squamous-cell invasion in nodes often results in necrosis or heterogeneity, with metastatic nodes exhibiting a signal intensity similar to that of the primary tumor. An irregular nodal outline or spiculated border, round shape, and strong nodal enhancement have also been reported to be useful features that suggest nodal involvement [11,48].

The location of nodal metastases in anal carcinomas depends on whether the primary tumor is located below or above the dentate line. Primary tumors located below the dentate line often involve the external iliac, inguinal, and deep inguinal nodes, whereas those located above the dentate line are more likely to have mesorectal and internal iliac nodal involvement. However, tumors extending across the dentate line can metastasize to any of these nodal regions.

**Figure 6:**
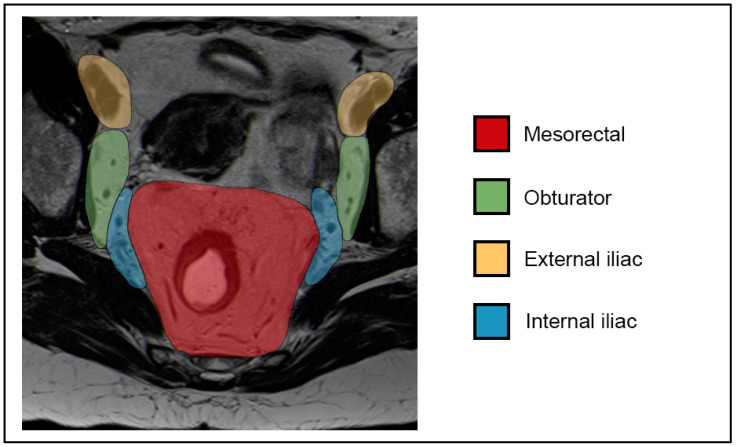
T2-weighted axial MR image: mesorectal, obturator, external, and internal iliac lymph node compartments.

**Figure 7:**
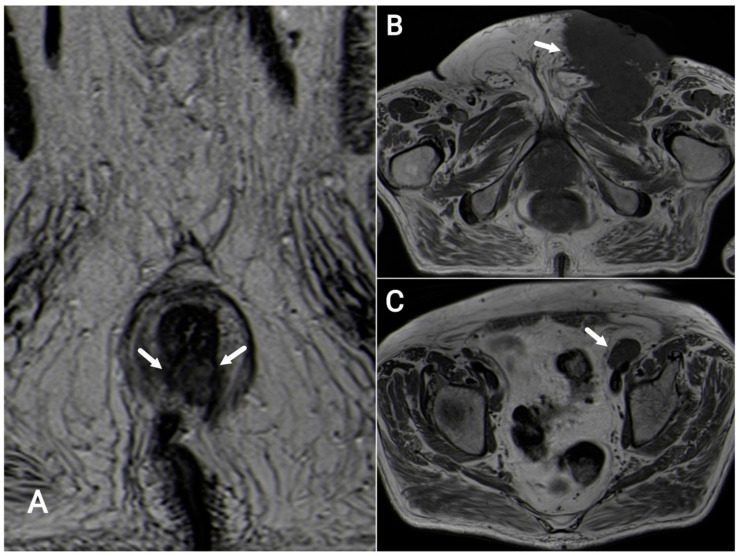
Squamous-cell carcinoma of the anal canal. Axial T2-weighted MR image (**A**, white arrows): 26 mm lesion from 5 to 7 o’clock invading the internal sphincter muscle bilaterally. Axial T1-weighted images: left inguinal adenopathy with cutaneous anterior extension (**B**); left external iliac adenopathy (**C**, white arrows).

The 8th edition of the AJCC guidelines for TNM staging of anal cancers, published in 2016, introduced notable changes in nodal staging. In the previous TNM 7th edition, lymph node status was classified as N0 and N1–N3. In the current TNM8, the N2 and N3 categories were eliminated, while the N1 category was further subdivided into N1a, N1b, and N1c [49]. In the TNM8, N0 indicates the absence of regional nodal metastases. N1a includes malignant lymph nodes located in the inguinal, mesorectal, or internal iliac regions. N1b corresponds to metastasis in the external iliac lymph nodes, whereas N1c refers to the presence of metastases in both the external iliac and N1a nodes. The external iliac lymph nodes are recognized as a regional site of disease (N) in the current TNM staging system, whereas previously they could also be considered to be a site of distal metastasis (M). Lymph nodes in the obturator region are part of the internal iliac group and, therefore, classified as N1a (Figure 6 and Figure 7).

### 7.3. M-Staging

In the TNM classification, M0 indicates no distant metastasis and M1 refers to distant metastasis. CT scan with intravenous contrast is the preferred method for detecting distant metastatic disease [50,51]. However, a recent study showed that FDG PET/CT has a greater sensitivity than CT imaging, revealing additional sites of distant metastasis in 3–5% of cases [52].

**Figure 8:**
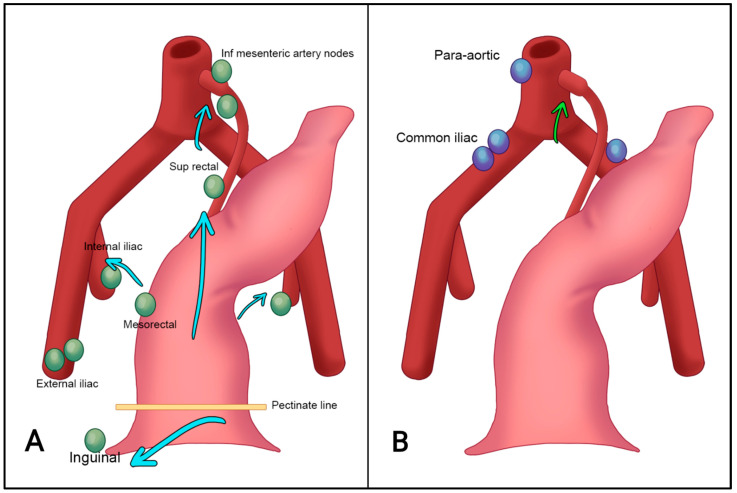
Anal cancer can spread to regional lymph nodes (N-staging) (**A**, green arrows) and non-regional lymph nodes (M-staging) (**B**).

According to the literature, approximately 6% of ASCCs are metastatic at diagnosis [40]. The most frequent locations of distant metastasis are the para-aortic and common iliac lymph nodes, as well as the liver and lungs [4,53,54] (Figure 8).

## 8. Treatment

Conventional treatment for ASCC involves chemoradiation therapy (CRT), which comprises pelvic radiation and concurrent chemotherapy (5-FU combined with mitomycin-c or cisplatin). CRT is the standard protocol, in contrast to rectal cancer, for which chemoradiation is used as neoadjuvant therapy prior to surgery [55,56]. This approach also permits sphincter preservation in most patients [21]. Imaging can also help in planning the radiation field and dosage and selecting the optimal surgical approach.

CRT has achieved a 5-year survival rate of roughly 80%, but in larger primary cancers (≥5 cm) the complete response rate may be lower (ranging from 50 to 75%) [21,57].

At present, wide local excision surgery with 1 cm margins of resection can be performed for SCCs located in the perianal area that are T1, node-negative, and well differentiated [5]. Anal margin (perianal skin) tumors are generally composed of keratinized squamous epithelium, tend to be more superficial, and grow slower than tumors located in other parts of the anal canal. If detected at an early stage (T1), these tumors may be more amenable to treatment with surgery rather than CRT.

Abdominoperineal resection may be necessary in patients with recurrent or residual tumors after chemoradiation [22].

### Evaluation after Chemoradiation Therapy

Around half of all relapses of ASCC that occur within two years after treatment are situated in the proximity of the primary site of disease, or in the perirectal, presacral, and inguinal lymph nodes and internal iliac node chains [58].

Although the guidelines of the NCCN, ESMO-ESSO-ESTRO, and the American Society of Colon and Rectal Surgery do not recommend the routine use of MRI for the evaluation of treatment response in patients with anal cancer, it is frequently employed in clinical settings for follow-up assessments. Specifically, MRI might be indicated for patients with initial T4 disease, those who display discrepancies between clinical examination and biopsy findings, and those with suspected recurrent tumors. In addition, imaging can assist in the diagnosis of certain complications associated with treatment, including fistula formation and insufficiency fractures [40,59,60].

Typically, post-chemoradiation evaluation using MRI is conducted 6–10 weeks after the last radiation dose. While a positive response is commonly observed at this stage, the possibility of residual tumor cannot be ruled out, since the maximal response may take up to six months to manifest after the conclusion of treatment [11,58]. The presence of treatment-related fibrosis may be more easily discernible from the intermediate signal intensity of the residual tumor 6 months after completing CRT [59]. Early identification of recurrence can be facilitated by carefully comparing the initial post-treatment baseline MRI with follow-up imaging.

A major challenge in using MRI is the high signal edema in the mucosa, which can result in a pseudotumor appearance due to CRT. This is particularly problematic to diagnose, as it is commonly accompanied by focal thickening, and anal cancer usually demonstrates a high signal on T2-weighted images [40]. DWI can aid in distinguishing a complete response from residual tumor [61,62]. Koh et al. reported that if the signal intensity on MRI remains stable one year after CRT, it may indicate prolonged complete remission [59]. Kochhar and colleagues introduced the concept of the “tram track sign” as a prospective marker for a complete response. This sign is characterized by the presence of low-signal bands between the inner and outer margins of the internal sphincter at the location of the previous tumor [63].

Patients with T3–T4 tumors or positive inguinal lymph nodes are recommended to undergo annual CT scans of the chest, abdomen, and pelvis for a period of three years. However, there are no official guidelines for post-CRT surveillance using EAUS, MRI, or PET/CT. FDG-PET may improve the diagnostic accuracy of evaluating relapses [5]. Nevertheless, extending the surveillance period to five years seems to be a reasonable approach [22].

Staging of the residual tumor can be performed using the same TNM stages that are used for primary staging [40].

## 9. Difference between Anal and Rectal Cancer TNM

The T1–T3 stages for anal cancer are based on size, while for rectal cancer they are based on the depth of invasion: T1–T2 tumors are confined to the intestinal wall, while T3 tumors extend into the perirectal fat [64].

It is necessary to distinguish between regional and non-regional lymph nodes. The internal iliac, obturator, and mesorectal lymph nodes, including those along the upper rectal venous and arterial branches, and the inferior mesenteric artery, are regional lymph nodes for rectal and anal cancers. The main difference is that the external iliac and inguinal lymph nodes are regional lymph nodes in anal cancer and distant metastases in rectal cancer. Common iliac and para-aortic lymph nodes are considered to be distant metastases for both.

The staging of regional lymph nodes is quite different. In anal cancer it is based on the location of the positive lymph nodes, while in rectal cancer it is based on the number of positive lymph nodes. For rectal cancer, according to the TNM classification system, N1 is defined as having up to three regional metastatic lymph nodes, and N2 is defined as having four or more metastatic lymph nodes [65]. 

The criteria to classify lymph nodes as malignant according to the ESGAR guidelines for rectal cancer [66] are as follows:

- Short-axis diameter ≥ 9 mm;
- Short-axis diameter 5–8 mm AND ≥2 morphologically suspicious characteristics *;
- Short-axis diameter < 5 mm AND 3 morphologically suspicious characteristics *;
- Mucinous lymph nodes (any size).

* Morphologically suspicious criteria:

- Round shape;
- Irregular border;
- Heterogeneous signal.

Regarding treatment for anal cancer, almost all patients receive CRT except for the early stages (T1N0). For rectal cancer, most patients undergo surgery, and neoadjuvant therapy and surgery are reserved for intermediate- and high-risk cases. Patients with distant metastases are not radically treatable and receive systemic therapy rather than locoregional therapy.

### Difference in Staging in Anal Cancer from TNM7 to TNM8

Compared with TNM7, TNM8 introduces more stage subcategories. Stage II is divided into IIA and IIB based on the T stage, whereas stage III is further categorized into IIIA, IIIB, and IIIC, based on both the T and N stages. Notably, patients classified as AJCC stage IIB under TNM8 are considered to be at a higher risk and may benefit from more aggressive or innovative treatment approaches [67].

## 10. Summary

The histology of the tumor before TNM evaluation is essential for proper staging through imaging.

The TNM classification system has multiple purposes. First, it defines the extent of the tumor at the time of diagnosis, which is crucial for selecting appropriate therapies and developing personalized management strategies. Second, it is a prognostic tool for predicting the disease outcome. Finally, it enables the comparison of different patients, which can aid in improving our knowledge of the disease and in guiding treatment decisions. Imaging also plays an essential role in response assessment and long-term follow-up.

MRI is considered to be the imaging technique of choice for evaluating the stage of anal cancers, assessing treatment response following CRT, and identifying potential complications, because of its ability to provide high contrast and precise anatomical resolution of the anal canal. Changes in tumor size, morphology, and signal intensity on MRI can indicate the effectiveness of treatment and guide further management decisions. Other imaging modalities, such as CT and PET, are used to evaluate anal canal cancer.

In the future, the use of AI and radiomics could improve staging, response evaluation, and surveillance, leading to improved treatment outcomes and patient survival; however, additional research is needed.

## 11. Example Report for Baseline Staging of Anal Cancer [68]

Location:

- Perianal skin vs. anal canal (cranial or caudal half);
- Circumferential position + degree of sphincter involvement;
- Extension into the rectum.

Size: largest measure in any plane:

- It is not possible to evaluate the primary tumor/no primary tumor can be identified (Tx/T0);
- ≤2 cm (T1);
- >2 and ≤5 cm (T2);
- >5 cm (T3).

Organ invasion: T4 (structures with invasion or possible invasion; does not include sphincter or pelvic floor, rectum, or skin).

DWI/ADC: restricted/no restricted diffusion.

Location of nodes:

- Inguinal, mesorectal, internal iliac/obturator (N1a);
- External iliac (N1b);
- Both N1a and N1b (N1c);
- Common iliac, para-aortic (M1).

Other: complications (e.g., fistulas), pelvic metastases, bones, and other pelvic organs.

## Data Availability

All data are reported in the manuscript.

## References

[1] Siegel R., Werner R.N., Koswig S., Gaskins M., Rödel C., Aigner F. (2021). Anal cancer—Diagnosis, treatment and follow-up. Dtsch. Arztebl. Int..

[2] Cattapan K., Chulroek T., Wancharoenrung D., Kordbacheh H., Harisinghani M. (2019). Can MR imaging be useful in differentiating low rectal cancer from anal cancer?. Abdom. Radiol..

[3] Nelson R.A., Levine A.M., Bernstein L., Smith D.D., Lai L.L. (2013). Changing patterns of anal canal carcinoma in the United States. J. Clin. Oncol..

[4] Golia Pernicka J.S., Sheedy S.P., Ernst R.D., Minsky B.D., Ganeshan D., Rauch G.M. (2019). MR staging of anal cancer: What the radiologist needs to know. Abdom. Radiol..

[5] Hemachandran N., Goyal A., Bhattacharjee H.K., Sharma R. (2021). Radiology of anal and lower rectal cancers. Clin. Radiol..

[6] Pessia B., Romano L., Giuliani A., Lazzarin G., Carlei F., Schietroma M. (2020). Squamous cell anal cancer: Management and therapeutic options. Ann. Med. Surg..

[7] Islami F., Ferlay J., Jemal A. (2017). International trends in anal cancer incidence rates. Int. J. Epidemiol..

[8] Young A.N., Jacob E., Willauer P., Smucker L., Monzon R., Oceguera L. (2020). Anal Cancer. Surg. Clin. N. Am..

[9] Mahmud A., Poon R., Jonker D. (2017). PET imaging in anal canal cancer: A systematic review and meta-analysis. Br. J. Radiol..

[10] Durot C., Dohan A., Boudiaf M., Servois V., Soyer P., Hoeffel C. (2017). Cancer of the anal canal: Diagnosis, staging and follow-up with MRI. Korean J. Radiol..

[11] Ciombor K.K., Ernst R.D., Brown G. (2017). Diagnosis and Diagnostic Imaging of Anal Canal Cancer. Surg. Oncol. Clin. N. Am..

[12] Erden A. (2018). MRI of anal canal: Normal anatomy, imaging protocol, and perianal fistulas: Part 1. Abdom. Radiol..

[13] Iacobellis F., Di Serafino M., Brillantino A., Mottola A., Del Giudice S., Stavolo C., Festa P., Patlas M.N., Scaglione M., Romano L. (2021). Role of MRI in early follow-up of patients with solid organ injuries: How and why we do it?. Radiol. Medica.

[14] Chiloiro G., Cusumano D., de Franco P., Lenkowicz J., Boldrini L., Carano D., Barbaro B., Corvari B., Dinapoli N., Giraffa M. (2022). Does restaging MRI radiomics analysis improve pathological complete response prediction in rectal cancer patients? A prognostic model development. Radiol. Medica.

[15] Shannon B.A., Ahlawat S., Morris C.D., Levin A.S., Fayad L.M. (2022). Do contrast-enhanced and advanced MRI sequences improve diagnostic accuracy for indeterminate lipomatous tumors?. Radiol. Medica.

[16] Petralia G., Zugni F., Summers P.E., Colombo A., Pricolo P., Grazioli L., Colagrande S., Giovagnoni A., Padhani A.R., Italian Working Group on Magnetic Resonance (2021). Whole-body magnetic resonance imaging (WB-MRI) for cancer screening: Recommendations for use. Radiol. Medica.

[17] Albano D., Stecco A., Micci G., Sconfienza L.M., Colagrande S., Reginelli A., Grassi R., Carriero A., Midiri M., Lagalla R. (2021). Whole-body magnetic resonance imaging (WB-MRI) in oncology: An Italian survey. Radiol. Medica.

[18] Wang Q., Xu J., Wang A., Chen Y., Wang T., Chen D., Zhang J., Brismar T.B. (2023). Systematic review of machine learning-based radiomics approach for predicting microsatellite instability status in colorectal cancer. Radiol. Medica.

[19] Danti G., Flammia F., Matteuzzi B., Cozzi D., Berti V., Grazzini G., Pradella S., Recchia L., Brunese L., Miele V. (2021). Gastrointestinal neuroendocrine neoplasms (GI-NENs): Hot topics in morphological, functional, and prognostic imaging. Radiol. Medica.

[20] Di Serafino M., Vallone G. (2021). The role of point of care ultrasound in radiology department: Update and prospective. A statement of Italian college ultrasound. Radiol. Medica.

[21] Glynne-Jones R., Nilsson P.J., Aschele C., Goh V., Peiffert D., Cervantes A., Arnold D., ESMO, ESSO, ESTRO (2014). Anal cancer: ESMO-ESSO-ESTRO clinical practice guidelines for diagnosis, treatment and follow-up. Radiother. Oncol..

[22] Stewart D.B., Gaertner W.B., Glasgow S.C., Herzig D.O., Feingold D., Steele S.R., Prepared on Behalf of the Clinical Practice Guidelines Committee of the American Society of Colon and Rectal Surgeons (2018). The American society of colon and rectal surgeons clinical practice guidelines for anal squamous cell cancers (Revised 2018). Dis. Colon Rectum.

[23] Moureau-Zabotto L., Vendrely V., Abramowitz L., Borg C., Francois E., Goere D., Huguet F., Peiffert D., Siproudhis L., Ducreux M. (2017). Anal cancer: French Intergroup Clinical Practice Guidelines for diagnosis, treatment and follow-up (SNFGE, FFCD, GERCOR, UNICANCER, SFCD, SFED, SFRO, SNFCP). Dig. Liver Dis..

[24] Chulroek T., Kordbacheh H., Wangcharoenrung D., Cattapan K., Heidari P., Harisinghani M.G. (2019). Comparative accuracy of qualitative and quantitative 18F-FDG PET/CT analysis in detection of lymph node metastasis from anal cancer. Abdom. Radiol..

[25] Granata V., Faggioni L., Grassi R., Fusco R., Reginelli A., Rega D., Maggialetti N., Buccicardi D., Frittoli B., Rengo M. (2022). Structured reporting of computed tomography in the staging of colon cancer: A Delphi consensus proposal. Radiol. Medica.

[26] Vicini S., Bortolotto C., Rengo M., Ballerini D., Bellini D., Carbone I., Preda L., Laghi A., Coppola F., Faggioni L. (2022). A narrative review on current imaging applications of artificial intelligence and radiomics in oncology: Focus on the three most common cancers. Radiol. Medica.

[27] Coppola F., Faggioni L., Regge D., Giovagnoni A., Golfieri R., Bibbolino C., Miele V., Neri E., Grassi R. (2021). Artificial intelligence: Radiologists’ expectations and opinions gleaned from a nationwide online survey. Radiol. Medica.

[28] Gurgitano M., Angileri S.A., Rodà G.M., Liguori A., Pandolfi M., Ierardi A.M., Wood B.J., Carrafiello G. (2021). Interventional Radiology ex-machina: Impact of Artificial Intelligence on practice. Radiol. Medica.

[29] Zerunian M., Pucciarelli F., Caruso D., Polici M., Masci B., Guido G., De Santis D., Polverari D., Principessa D., Benvenga A. (2022). Artificial intelligence based image quality enhancement in liver MRI: A quantitative and qualitative evaluation. Radiol. Medica.

[30] Scapicchio C., Gabelloni M., Barucci A., Cioni D., Saba L., Neri E. (2021). A deep look into radiomics. Radiol. Medica.

[31] Nardone V., Reginelli A., Grassi R., Boldrini L., Vacca G., D’Ippolito E., Annunziata S., Farchione A., Belfiore M.P., Desideri I. (2021). Delta radiomics: A systematic review. Radiol. Medica.

[32] Flammia F., Innocenti T., Galluzzo A., Danti G., Chiti G., Grazzini G., Bettarini S., Tortoli P., Busoni S., Dragoni G. (2023). Branch duct-intraductal papillary mucinous neoplasms (BD-IPMNs): An MRI-based radiomic model to determine the malignant degeneration potential. Radiol. Medica.

[33] Shen L.-L., Zheng H.-L., Ding F.-H., Lu J., Chen Q.-Y., Xu B.-B., Xue Z., Lin J., Huang C.-M., Zheng C.-H. (2023). Delta computed tomography radiomics features-based nomogram predicts long-term efficacy after neoadjuvant chemotherapy in advanced gastric cancer. Radiol. Medica.

[34] Benedetti G., Mori M., Panzeri M.M., Barbera M., Palumbo D., Sini C., Muffatti F., Andreasi V., Steidler S., Doglioni C. (2021). CT-derived radiomic features to discriminate histologic characteristics of pancreatic neuroendocrine tumors. Radiol. Medica.

[35] Palatresi D., Fedeli F., Danti G., Pasqualini E., Castiglione F., Messerini L., Massi D., Bettarini S., Tortoli P., Busoni S. (2022). Correlation of CT radiomic features for GISTs with pathological classification and molecular subtypes: Preliminary and monocentric experience. Radiol. Medica.

[36] Karmazanovsky G., Gruzdev I., Tikhonova V., Kondratyev E., Revishvili A. (2021). Computed tomography-based radiomics approach in pancreatic tumors characterization. Radiol. Medica.

[37] Cozzi D., Bicci E., Cavigli E., Danti G., Bettarini S., Tortoli P., Mazzoni L.N., Busoni S., Pradella S., Miele V. (2022). Radiomics in pulmonary neuroendocrine tumours (NETs). Radiol. Medica.

[38] Santone A., Brunese M.C., Donnarumma F., Guerriero P., Mercaldo F., Reginelli A., Miele V., Giovagnoni A., Brunese L. (2021). Radiomic features for prostate cancer grade detection through formal verification. Radiol. Medica.

[39] Xue K., Liu L., Liu Y., Guo Y., Zhu Y., Zhang M. (2022). Radiomics model based on multi-sequence MR images for predicting preoperative immunoscore in rectal cancer. Radiol. Medica.

[40] Maas M., Tielbeek J.A.W., Stoker J. (2020). Staging of Anal Cancer: Role of MR Imaging. Magn. Reson. Imaging Clin. N. Am..

[41] Matalon S., Mamon H., Rosenthal M. (2015). Anorectal cancer: Critical anatomic and staging distinctions that affect use of radiation therapy. Radiographics.

[42] Rosa C., Caravatta L., Di Tommaso M., Fasciolo D., Gasparini L., Di Guglielmo F.C., Augurio A., Vinciguerra A., Vecchi C., Genovesi D. (2021). Cone-beam computed tomography for organ motion evaluation in locally advanced rectal cancer patients. Radiol. Medica.

[43] Touboul E., Schlienger M., Buffat L., Lefkopoulos D., Pène F., Parc R., Tiret E., Gallot D., Malafosse M., Laugier A. (1994). Epidermoid carcinoma of the anal canal. Results of curative-intent radiation therapy in a series of 270 patients. Cancer.

[44] Benson A.B., Venook A.P., Al-Hawary M.M., Cederquist L., Chen Y.J., Ciombor K.K., Cohen S., Cooper H.S., Deming D., Engstrom P.F. (2018). Anal Carcinoma, version 2.2018 clinical practice guidelines in Oncology. JNCCN J. Natl. Compr. Cancer Netw..

[45] Jones M., Hruby G., Solomon M., Rutherford N., Martin J. (2015). The Role of FDG-PET in the Initial Staging and Response Assessment of Anal Cancer: A Systematic Review and Meta-analysis. Ann. Surg. Oncol..

[46] Sandach P., Kasper-Virchow S., Rischpler C., Herrmann K. (2020). Molecular Imaging and Therapy of Colorectal and Anal Cancer. Semin. Nucl. Med..

[47] Scialpi M., Moschini T.O., De Filippis G. (2022). PET/contrast-enhanced CT in oncology: “to do, or not to do, that is the question”. Radiol. Medica.

[48] Parikh J., Shaw A., Grant L.A., Schizas A.M., Datta V., Williams A.B., Griffin N. (2011). Anal carcinomas: The role of endoanal ultrasound and magnetic resonance imaging in staging, response evaluation and follow-up. Eur. Radiol..

[49] Amin M.B., Greene F.L., Edge S.B., Compton C.C., Gershenwald J.E., Brookland R.K., Meyer L., Gress D.M., Byrd D.R., Winchester D.P. (2017). The Eighth Edition AJCC Cancer Staging Manual: Continuing to build a bridge from a population-based to a more “personalized” approach to cancer staging. CA Cancer J. Clin..

[50] Masci G.M., Ciccarelli F., Mattei F.I., Grasso D., Accarpio F., Catalano C., Laghi A., Sammartino P., Iafrate F. (2022). Role of CT texture analysis for predicting peritoneal metastases in patients with gastric cancer. Radiol. Medica.

[51] Rampado O., Depaoli A., Marchisio F., Gatti M., Racine D., Ruggeri V., Ruggirello I., Darvizeh F., Fonio P., Ropolo R. (2021). Effects of different levels of CT iterative reconstruction on low-contrast detectability and radiation dose in patients of different sizes: An anthropomorphic phantom study. Radiol. Medica.

[52] Niyoteka S., Seban R.D., Rouhi R., Scarsbrook A., Genestie C., Classe M., Carré A., Sun R., La Greca Saint-Esteven A., Chargari C. (2023). A common [18F]-FDG PET radiomic signature to predict survival in patients with HPV-induced cancers. Eur. J. Nucl. Med. Mol. Imaging.

[53] Granata V., Fusco R., De Muzio F., Cutolo C., Setola S.V., Dell’Aversana F., Grassi F., Belli A., Silvestro L., Ottaiano A. (2022). Radiomics and machine learning analysis based on magnetic resonance imaging in the assessment of liver mucinous colorectal metastases. Radiol. Medica.

[54] Granata V., Fusco R., De Muzio F., Cutolo C., Setola S.V., Grassi R., Grassi F., Ottaiano A., Nasti G., Tatangelo F. (2022). Radiomics textural features by MR imaging to assess clinical outcomes following liver resection in colorectal liver metastases. Radiol. Medica.

[55] Cusumano D., Meijer G., Lenkowicz J., Chiloiro G., Boldrini L., Masciocchi C., Dinapoli N., Gatta R., Casà C., Damiani A. (2021). A field strength independent MR radiomics model to predict pathological complete response in locally advanced rectal cancer. Radiol. Medica.

[56] Fusco R., Granata V., Sansone M., Rega D., Delrio P., Tatangelo F., Romano C., Avallone A., Pupo D., Giordano M. (2021). Validation of the standardized index of shape tool to analyze DCE-MRI data in the assessment of neo-adjuvant therapy in locally advanced rectal cancer. Radiol. Medica.

[57] Wright J.L., Wright J.L., Patil S.M., Temple L.K., Minsky B.D., Saltz L.B., Goodman K.A. (2010). Squamous cell carcinoma of the anal canal: Patterns and predictors of failure and implications for intensity-modulated radiation treatment planning. Int. J. Radiat. Oncol. Biol. Phys..

[58] Gourtsoyianni S., Goh V. (2014). MRI of anal cancer: Assessing response to definitive chemoradiotherapy. Abdom. Imaging.

[59] Koh D.M., Dzik-Jurasz A., O’Neill B., Tait D., Husband J.E., Brown G. (2014). Pelvic phased-array MR imaging of anal carcinoma before and after chemoradiation. Br. J. Radiol..

[60] Pozzessere C., Boudiaf M., Cirigliano A., Dohan A., Mazzei M.A., Barat M., Volterrani L., Soyer P. (2022). MR-enterography: Role in the assessment of suspected anastomotic recurrence of Crohn disease after ileocolic resection. Radiol. Medica.

[61] Cicero G., Alibrandi A., Blandino A., Ascenti V., Fries W., Viola A., Mazziotti S. (2022). DWI ratios: New indexes for Crohn’s disease activity at magnetic resonance enterography?. Radiol. Medica.

[62] Gitto S., Bologna M., Corino V.D.A., Emili I., Albano D., Messina C., Armiraglio E., Parafioriti A., Luzzati A., Mainardi L. (2022). Diffusion-weighted MRI radiomics of spine bone tumors: Feature stability and machine learning-based classification performance. Radiol. Medica.

[63] Kochhar R., Renehan A.G., Mullan D., Chakrabarty B., Saunders M.P., Carrington B.M. (2017). The assessment of local response using magnetic resonance imaging at 3- and 6-month post chemoradiotherapy in patients with anal cancer. Eur. Radiol..

[64] Horvat N., Rocha CC T., Oliveira B.C., Petkovska I., Gollub M.J. (2019). MRI of rectal cancer: Tumor staging, imaging techniques, and management. Radiographics.

[65] Beets-Tan R.G.H., Lambregts D.M.J., Maas M., Bipat S., Barbaro B., Curvo-Semedo L., Fenlon H.M., Gollub M.J., Gourtsoyianni S., Halligan S. (2018). Magnetic resonance imaging for clinical management of rectal cancer: Updated recommendations from the 2016 European Society of Gastrointestinal and Abdominal Radiology (ESGAR) consensus meeting. Eur. Radiol..

[66] Zhao L., Liang M., Wu P.Y., Yang Y., Zhang H., Zhao X. (2021). A preliminary study of synthetic magnetic resonance imaging in rectal cancer: Imaging quality and preoperative assessment. Insights Imaging.

[67] Goffredo P., Garancini M., Robinson T.J., Frakes J., Hoshi H., Hassan I. (2018). A National-Level Validation of the New American Joint Committee on Cancer 8th Edition Subclassification of Stage IIA and B Anal Squamous Cell Cancer. Ann. Surg. Oncol..

[68] Kassam Z., Lang R., Arya S., Bates D.D.B., Chang K.J., Fraum T.J., Friedman K.A., Golia Pernicka J.S., Gollub M.J., Harisinghani M. (2022). Update to the structured MRI report for primary staging of rectal cancer: Perspective from the SAR Disease Focused Panel on Rectal and Anal Cancer. Abdom. Radiol..

